# A Review of the Various Surface Treatments of NiTi Instruments

**Published:** 2014-10-07

**Authors:** Zahed Mohammadi, Mohammad Karim Soltani, Sousan Shalavi, Saeed Asgary

**Affiliations:** a *Iranian Center for Endodontic Research, Research Institute of Dental Sciences, Shahid Beheshti University of Medical Sciences, Tehran, Iran;*; b *Department of Orthodontics, Hamedan University of Medical Sciences, Hamedan, Iran;*; c *General Dental Practitioner, Hamedan, Iran*

**Keywords:** Cryogenic Treatment, Electropolishing, Ion Implantation, Nickel-Titanium, NiTi, Thermal Nitridation

## Abstract

Since the introduction of engine-driven nickel-titanium (NiTi) instruments, attempts have been made to minimize or eliminate their inherent defects, increase their surface hardness/flexibility and also improve their resistance to cyclic fatigue and cutting efficiency. The various strategies of enhancing instrument surface include ion implantation, thermal nitridation, cryogenic treatment and electropolishing. The purpose of this paper was to review the metallurgy and crystal characteristics of NiTi alloy and to present a general over review of the published articles on surface treatment of NiTi endodontic instruments.

## Introduction

Nickel-titanium (NiTi) instruments show much greater flexibility and resistance to torsional fracture in comparison with stainless steel (SS) instruments [1]. In addition, NiTi alloy has more modulus of elasticity (MOE), a wider range of elastic deformation and greater strength [1, 2]. However, due to pseudoelasticity of this alloy, manufacturing of the NiTi instruments must be done through machining rather than twisting a NiTi wire [3]. The machining process may lead to surface defects within the cutting surfaces of the as-received instrument, which *per se* result in relatively low cutting efficiency of the file and decreased resistance to fracture [3]. NiTi instruments also exhibit a lower microhardness (MH) (303–362 VHN) compared to SS files (522–542 VHN) [4, 5]. Thus, surface defects occur more frequently which leads to instrument wear. This combination of surface wear and lower MH decrease the cutting efficiency of NiTi files [6].

Improvement of the cutting efficiency of NiTi instruments especially through their surface treatment has been the subject of numerous investigations. The implantation of boron ions on the surface of the instruments increases their surface hardness [7]. Also, increased wear-resistance/cutting efficiency of NiTi was observed after thermal nitridation process [8] and physical vapor deposition of titanium nitride (Ti_3_N_4_) particles [6]. The aim of this article is to review the effect of various surface treatment strategies on the properties of NiTi files.

## Methods and Materials

***Retrieval of literature***

An English-limited Medline search was performed through the articles published from May 1988 to May 2014. The searched keywords included “electropolishing AND NiTi rotary instruments”, “thermal nitridation”, “cryogenic treatment AND nickel-titanium”, “plasma immersion ion implantation AND nickel-titanium”, and “endodontic treatment AND nickel-titanium”. Then, a hand search was done in the references of result articles to find more matching papers.

## Results

A total of 176 articles were found which according to their related keywords were “10-electropolishing AND NiTi rotary instruments”, “29-thermal nitridation”, “3-cryogenic treatment AND nickel-titanium”, “135-plasma immersion ion implantation AND nickel-titanium”, and “endodontic treatment AND nickel-titanium”.

***Metallurgy of NiTi***

The NiTi alloys used for manufacturing of the endodontic instruments contain approximately 56% (wt) nickel and 44% (wt) titanium. In some NiTi alloys, a small percentage (<2% wt) of nickel can be substituted by cobalt. The resultant combination is a 1:1 atomic ratio (equiatomic) of the major components. The generic term for these alloys is 55-nitinol and as other metallic systems, the alloy can exist in various crystallographic forms. NiTi has inherent ability of shape memory (SM) and super-elasticity (SE) [3] that stem from the ability to alter the type of atomic bonding which causes unique and significant changes in the mechanical properties and crystallographic arrangement of the alloy; the transition between the “austenite” and “martensite” phases in the NiTi alloy is a function of temperature and stress. 

The concept of SM was first described by Ölander [9] during evaluation of a cadmium-gold alloy. Beside SM, NiTi exhibits SE that enables it to return to its original shape upon unloading following substantial deformation. SE is a consequence of the reversible phase transformation between austenite and martensite phases and has a critical influence on the mechanical properties of the alloy, which can be readily altered by small changes in composition, impurities and heat treatment. This distinct property of NiTi alloy has caused a revolution in the manufacturing of endodontic instruments[3].

Under low tensile loading, the superelastic NiTi alloy shows a normal elastic behavior. At higher tensile loading, the elastic stress reaches plateau at which there is an extended horizontal region of elastic strain. Elastic deformation in SS and NiTi is 3% and 7%, respectively. The atoms in SS can move against each other by a small specific amount before plastic deformation occurs which is called the Hookean elasticity [10]. 

***Crystal characteristics of NiTi alloy***

All the alloys with SM show a change in their lattice structure or atomic arrangement, characterizing a phase change while receiving or releasing thermal energy. The critical deformation and shape recovery are explained according to the changes in the lattice parameters through a transformation between the austenite and martensite phases and the characteristics of the crystal structure [11]. At high temperature ranges the lattice of NiTi alloy is a body-centered cubic structure which is referred to as the austenite or parent phase. Upon cooling, NiTi structure goes through a critical-transformation change to R-phase and shows changes in its MOE (stiffness), yield strength, and electric resistivity. By more reduction in the temperature through this range, there is a change in the crystal structure which is known as the martensitic transformation; the amount of this transformation is a function of the start (M_s_) and finish (M_f_) temperature. Likewise the temperatures at which the austenite starts and finishes are called the A_s _and A_f_ points, respectively. The event causes alterations in the physical properties of the alloy and allows SM [11, 12, 13].

***Surface of NiTi instrument***

The surface of NiTi instrument mainly consists of oxygen, carbon, and titanium oxides (TiO_2_) and smaller amounts of nickel oxides (NiO and Ni_2_O_3_) and metallic nickel (Ni) [14-19]. The thickness of the oxide layer varies between 2-20 nm. Depending on the preparation method, the surface chemistry and the amount of Ni may vary in a wide range [20]. Ni may dissolve more easily than titanium (Ti) because its oxide is not so stable. Surface layers of NiTi arch wires have irregular features characterized by long island-like structures, which are indicative of selective dissolution of Ni [17].

Shabalovskaya [18] found that when the surface of wire was mechanically polished, the Ti:Ni ratio was 5.5, showing that the amount of Ti on the surface was five times more. When the wire was boiled or autoclaved in water, the concentration of Ni decreased and the Ti:Ni ratio increased to 23.4-33.1. The findings of Hanawa *et al.* [14] were similar. The Ti:Ni ratio was 5.8 in polished samples, but increased up to 91 when the sample was immersed for 30 days in a neutral electrolyte solution. In the study by Hanawa *et al.*, the surface of titanium-aluminum-vanadium alloy (Ti_6_Al_4_V) had amounts of aluminum similar to the amount of Ni in NiTi, even though the bulk material of Ti_6_Al_4_V had only 6% Al and NiTi had 50% Ni. There was no Ni on the surface of SS, but some amounts of Cr and Fe were found.

Pure Ti and some of its alloys are amongst the most biocompatible materials [20]. Their favorable biocompatibility is thought to be due to the stable titanium oxide layer. During placement of a Ti implant, the oxide layer formed on the implant grows and absorbs minerals and other constituents of tissue fluids and these reactions in turn cause remodeling of the surface. Hanawa [14] found that the oxide layer on the implants consists of two layers of calcium phosphate and TiO_2_. In other words, calcium phosphate formed on an inert oxide layer. This layer was thicker on pure titanium than on titanium alloys (including NiTi), and the Ca:P ratio of the film was close to that of hydroxyapatite. The calcium phosphates formed on NiTi or Ti_6_Al_4_V were less similar to hydroxyapatite. The presence of Ni in the surface of NiTi alloy and aluminum in the surface of Ti_6_Al_4_V may have caused these results. SS also has a calcium phosphate layer of this kind. However, the formation of this layer is slower and differs from NiTi [14, 21, 22].

***Surface treatment of NiTi***

Attempts to enhance the surface of NiTi instruments, minimize or eliminate their inherent defects, increase the surface hardness/flexibility and improve the resistance to cyclic fatigue and cutting efficiency of endodontic instruments have resulted in a variety of strategies [23]. 

***Plasma immersion ion implantation***

There have been several attempts to reduce the release of Ni from NiTi, without deteriorating the mechanical properties of the bulk. This has been done by coating technologies either with titanium nitride (TiN) or with polymers [24]. The polymer coating is not suitable for many medical especially orthopedic implants. On the other hand, the hard TiN coatings frequently have disadvantages due to the interface between the bulk and its coating. Ion implantation can solve this problem, as a continuous interface between the surface and the bulk is created [31].

**Table 1:** Key studies on the ion implantation (II) of NiTi rotary instruments

**Authors **	**Results **
**Gavini ** ***et al.*** ** [** 25 **]**	Ion-implanted instruments reached significantly higher cycle numbers before fracture compared to annealed and non-implanted files.
**Wolle** *** et al. *** **[** 26 **]**	Argon II improved the performance of S1 files moderately, whereas nitrogen ion-implanted files performed worse in the fatigue test.

**Table 2:** Key studies on the thermal nitridation (TN) of NiTi rotary instruments

**Authors**	**Results**
**Shenhar ** ***et al. *** **[** 27 **]**	TN improved the corrosion resistance of Ti and/or Ti alloys in corrosive environments.
**Huang ** ***et al.*** ** [** 28 **]**	TN improved the corrosion resistance of Ti and/or Ti alloys in corrosive environments.
**Li ** ***et al.*** ** [** 29 **]**	TN of NiTi instruments at various temperatures increased cutting efficiency and corrosion resistance.
**Lin ** ***et al.*** ** [** 30 **]**	TN significantly increased the corrosion resistance.

Plasma immersion ion implantation (PIII) was first introduced in the late 1980s by Conrad *et al.* [32] and Tendys *et al. *[33]. This technique was first called plasma source ion implantation (PSII) and later was referred to as plasma-based ion implantation (PBII). The plasma can be generated by a number of methods such as hot filament discharge, inductively coupled plasma, radio frequency, electron cyclotron resonance system or high voltage bias self-ignition [33].

During PIII, the specimen is placed in a chamber and immersed in the plasma; then a highly negative pulsating voltage is applied to the sample. PIII is regularly performed to modify the surface of metals and to improve the mechanical properties such as hardness, friction coefficients, and wear/corrosion resistance [24, 31]. Briefly, ion implantation is a line-of-sight process in which ions are extracted from plasma, accelerated, and bombarded into a device.

Gavini *et al.* [25] showed that nitrogen ion implantation improved the cyclic fatigue resistance of NiTi rotary instruments. They found that ion-implanted instruments reached significantly higher value of CTF (cycles to fracture) (510 cycles) compared with annealed (428 cycles) and non-implanted files (381 cycles). Wolle *et al.* [26] evaluated the effect of nitrogen and argon ion implantation on morphologic alterations and fatigue resistance of S1 ProTaper rotary instruments. Their findings showed that argon implantation caused a moderate improvement in the performance of S1 files whereas nitrogen ion-implanted files showed a weaker performance in the fatigue test. They attributed the reduction in file performance to nitrogen diffusion in the grain boundaries, instead of the desired improvement caused by titanium nitride (TiN) formation [26] (Table 1).

***Thermal nitridation***

TiN belongs to the refractory transition metal family [34] and consists of both covalent and metallic bonds [25, 26]. The nitriding method known as powder immersion reaction assisted coating (PIRAC) produces TiN on NiTi [35]. The process is as follows: NiTi samples with a phase transform temperature at A_f_=15^°^C are annealing at 900^°^C for 1.5 h and then 1000^°^C for 1 h in sealed containers. Nitrogen (N) atoms diffuse into the samples and atmospheric oxygen is stopped by a steel foil consisting of a notable amount of Cr. The modified surface consists of a thin outer layer of TiN and a thicker Ti_2_Ni layer underneath [34, 35].

Shenhar *et al.* [27] and Huang *et al.* [28] showed that the TiN layer improved the corrosion resistance of Ti and its alloys in corrosive environments. Li *et al.* [29] showed that nitriding surface treatment of NiTi instruments at various temperatures increased the cutting efficiency and corrosion resistance in contact with sodium hypochlorite (NaOCl). In another study, Lin *et al.* [30] revealed that the placement of a TiN layer on commercial rotary NiTi instruments at 200˚C, 250˚C and 300˚C significantly increased the corrosion resistance of files placed in contact with 5.25% NaOCl. Although the files nitrided at 300˚C showed the highest polarization resistance and lowest passive current, the clinical application of this method is not recommended as at this temperature the SE character of the instrument may be lost. Therefore, the instruments nitrided at 250˚C are preferred for clinical application [30] (Table 2).

***Cryogenic treatment***

The cold treatment of metals during manufacturing is advocated so as to improve the surface hardness and thermal stability of the metal [36]. The optimum temperature range for cold treatment is between -60˚C and -80^°^C depending upon the material and the quenching parameters involved [36]. For the past 30 years, researchers have reported substantial benefits from subjecting metals for industrial applications to a cryogenic process [36-38]. 

As a newer cooling approach, cryogenic treatment (CT) involves submersing metal in a super-cooled bath containing liquid N (-196˚C/-320˚F) [36, 37] and then allowing the metal to slowly warm at room temperature [38, 39]. CT has more optimal effects than cold treatment [40]. The benefits include increasing the cutting efficiency as well as the overall strength of the metal [36, 38]. CT is an inexpensive treatment that affects the entire cross-section of the metal rather than just the surface as in surface treatment techniques including ion implantation and vapor deposition [37]. 

**Table 3:** Studies on the cryogenic treatment (CT) of NiTi rotary instruments

**Authors**	**Results **
**Kim ** ***et al.*** **[**41**]**	Cryogenically treated specimens had a significantly higher microhardness than the controls.
**Vinothkumar ** ***et al.*** **[**42**]**	Deep dry CT increased the cutting efficiency of NiTi instruments, significantly.
**George ** ***et al.*** **[**43**]**	Deep CT improved the cyclic fatigue resistance of NiTi rotary files, significantly.

**Table 4:** Studies on the electropolishing (EP) of NiTi rotary instruments

**Authors **	**Results **
**Herold ** ***et al.*** **[**49**]**	EP did not inhibit the development of microfractures in EndoSequence rotary instruments.
**Anderson ** ***et al.*** **[**46**]**	EP prolonged the fatigue life of rotary NiTi endodontic instruments.
**Bui ** ***et al.*** **[**50**]**	EP significantly reduced resistance to cyclic fatigue but did not affect torsional resistance and cutting efficiency, however, the angle at failure and amount of unwinding were decreased.
**Cheung ** ***et al.*** **[**48**]**	EP did not enhance low cyclic fatigue life of NiTi instruments subjected to rotational bending. In addition, EP did not improve the resistance to corrosion of strain-cycled instruments.
**Boessler ** ***et al.*** **[**51**]**	EP of ProTaper shaping instruments led to increased torque during preparation of simulated root canals.
**Tripi ** ***et al.*** **[**52**]**	The electropolished race instrument demonstrates an increased resistance to fatigue failure.
**Praisarnti ** ***et al.*** **[**53**]**	Low cyclic fatigue failure of a RaCe NiTi instrument rotating with a curvature in a corrosive environment enhanced by a magneto EP process.
**Lopes ** ***et al.*** **[**54**]**	The EP of BioRace endodontic instruments significantly increased the number of cycles to fracture under rotating-bending conditions within an artificial curved canal.

Two mechanisms can change the properties of metal during CT. First is a more complete martensite transformation from the austenite phase following CT [39] and second is the precipitation of finer carbide particles within the crystalline structure [38]. Controversy exists about which mechanism to be the main one. 

There are few studies on the CT of NiTi rotary instruments. Kim *et al.* [41] evaluated the effects of cryogenic treatment on the composition, microhardness or cutting efficiency of NiTi rotary instruments. According to their findings, cryogenically treated instruments had significantly higher microhardness than the controls. Furthermore, both test and control groups were composed of 56% wt Ni, 44% wt Ti and 0% N with a majority being in the austenite phase. In another study, Vinothkumar *et al.* [42] showed that deep dry CT significantly increased the cutting efficiency of NiTi instruments but it was not effective on the wear resistance. Also George *et al.* [43] reported that deep CT significantly improved the cyclic fatigue resistance of NiTi rotary files (Table 3). 

***Electropolishing***

Electropolishing (EP), *aka* electrochemical polishing, electro-lytic polishing and reverse plating, is an electrochemical process for removal of the material layer from a metallic surface. It often acts against electroplating. It may be used *in lieu* of abrasive fine polishing during microstructural preparation [44]. 

EP is a standard surface treatment process employed as a final finish during manufacturing of NiTi instruments. An electric potential and current are applied, which result in ionic dissolution of the surface. In this process, the surface chemistry and morphology are altered while surface imperfections are removed as dissolved metal ions. Simultaneously, Ti is oxidized to TiO_2_, which protects the underlying material from further corrosion. Generally, EP removes the native oxide layer and sinters a more homogeneous and stable passive TiO_2_ layer. In this process, the amount of Ni on the surface decreases [45-47].

Typically, the instrument is immersed in a temperature-controlled bath of electrolyte and serves as the anode when it is connected to the positive terminal of a direct current power supply, and the negative terminal is attached to the cathode. As the current passes the surface of metal oxidizes and dissolves in the electrolyte. At the cathode, a reduction reaction occurs, which normally produces hydrogen. Electrolytes used for EP are most often concentrated acid solutions with a high viscosity, such as mixtures of sulfuric/phosphoric acid. Other EP electrolytes include mixtures of perchlorates with acetic anhydride and methanolic solutions of sulfuric acid [45, 46].

EP of NiTi instruments is efficient for the elimination of defective surface layers through surface oxidizing. Owing to a gain in total energy caused by the differences in the enthalpy of Ti and Ni oxides forming, the preferential oxidation of Ti on NiTi surface always occurs [48]. Therefore, depending on the electrolytes and regimes employed, bare NiTi surfaces are built from Ti oxides with Ni concentrations from 2% to 7%. EP of NiTi can be used for surface structuring as well [3]. 

Using scanning electron microscopy (SEM), Herold *et al.* [49] showed that EP did not inhibit the development of micro-fractures in EndoSequence rotary instruments. According to Anderson *et al.* [46] EP may have beneficial effects in prolonging the fatigue life of NiTi endodontic instruments. They attributed the benefits of EP to the reduction in surface irregularities that serves as points for stress concentration and crack initiation. Bui *et al. *[50] investigated the effect of EP on torque resistance, fatigue resistance and cutting efficiency of ProFile instruments. Results revealed that EP significantly reduced the file’s resistance to cyclic fatigue but did not affect torsional resistance and cutting efficiency.

However, the torque at failure and amount of unwinding were decreased. Cheung *et al.* [48] showed that the low cyclic fatigue life of NiTi instruments subjected to rotational bending was not enhanced by EP. They further reported that EP did not improve the resistance to corrosion of strain-cycled files [48]. Boessler *et al.* [51] showed that EP of ProTaper Shaping instruments led to increased torque during preparation of simulated root canals. Tripi *et al.* [52] demonstrated that the EP RaCe instruments have increased resistance to fatigue failure, compared with ProFile, K3, HERO and Mtwo instruments. Praisarnti *et al.* [53] showed that low cyclic fatigue failure of RaCe instrument rotating in a curved canal and a corrosive environment, was enhanced by an EP process. Lopes *et al.* [54] revealed that the EP surface treatment of BioRace instruments significantly increased the number of CTF under rotating-bending conditions within an artificial curved canal. Condorelli *et al.* [55] showed that EP improved the resistance of instruments against fatigue fracture (Table 4).

## Conclusions

1. Argon implantation caused a moderate improvement in the performance of S1 files, whereas nitrogen ion-implanted files performed worse in the fatigue test. Furthermore, nitrogen ion implantation improves the cyclic fatigue of instruments.

2. Thermal nitridation increases the cutting efficiency and corrosion resistance of NiTi files in contact with NaOCl. 

3. Cryogenic treatment improves the cutting efficiency, cyclic fatigue resistance and microhardness of NiTi instruments.
